# First Autochthonous Infection of a Cat with *Dirofilaria immitis* in Austria

**DOI:** 10.3390/pathogens10091104

**Published:** 2021-08-30

**Authors:** Lisa-Maria Kulmer, Maria Sophia Unterköfler, Hans-Peter Fuehrer, Varvara Janovska, Matus Pagac, Michaela Svoboda, Luigi Venco, Michael Leschnik

**Affiliations:** 1 University Hospital for Small Animals, Department for Companion Animals and Horses, University of Veterinary Medicine Vienna, 1210 Vienna, Austria; michael.leschnik@vetmeduni.ac.at; 2 Department of Pathobiology, Institute of Parasitology, University of Veterinary Medicine Vienna, 1210 Vienna, Austria; maria.unterkoefler@vetmeduni.ac.at (M.S.U.); Hans-Peter.Fuehrer@vetmeduni.ac.at (H.-P.F.); 3 Veterinary Hospital Parndorf, 7111 Burgenland, Austria; varvara.janovska@gmail.com (V.J.); matus.pagac@gmail.com (M.P.); Michaela.svoboda@hotmail.com (M.S.); 4 Veterinary Hospital Città di Pavia, 27100 Pavia, Italy; luigivenco@libero.it

**Keywords:** dirofilariosis, feline heartworm disease, vector borne, caval syndrome, heartworm associated respiratory disease

## Abstract

This case report is about a seven-year-old male neutered European Shorthair cat infected by *Dirofilaria immitis* as the first reported autochthonous *Dirofilaria immitis* infection in Austria. There was no history of periods abroad. Echocardiography showed suspected *D. immitis* in the right cardiac chamber with increased pulmonary pressure and ascites. Surgical removal of the heartworms was performed. Twenty adult heartworms were removed by transvenous jugular approach under general anesthesia and stored in 4% formalin. Five out of 20 specimens were examined via light and stereomicroscopy and feline heartworm infection was confirmed. Amplification of a 203 bp or 724 bp fragment of the cytochrome c oxidase subunit I gene was unsuccessful. After surgery the cat developed acute renal failure but recovered quickly. One year later, the cat underwent a control examination including echocardiography and blood work. There were no more *D. immitis* detectable at echocardiography. Lung pressure was mildly increased. Complete blood count and creatinine were unremarkable. The Knott’s test and *Dirofilaria*-Antigen-test produced negative results. The cat did not show any clinical signs during the follow-up period. The aim of this case report is to highlight the growing risk of acquiring infection with *D. immitis* not only for Austrian dogs, but also for cats. This case report represents the first report of autochthonous *D. immitis* infection in Austria. Moreover, even if the prognosis in cats with caval syndrome due to feline heartworm disease is guarded to poor, surgical removal of the filariae can be a successful treatment option.

## 1. Introduction

Feline heartworm disease can be quite a challenging diagnosis for veterinarians due to its unique nature and physiopathology [1]. *Dirofilaria immitis* belongs to filarioid nematodes and represents the underlying agent for feline heartworm disease (FHWD) and heartworm associated respiratory disease (HARD). *D. immitis* infects mainly dogs but also cats, ferrets, wild carnivores and humans, and more than 70 different species of culicid mosquitos can act as vectors [2,3]. *D. immitis* infection has been reported mainly in temperate, tropical and subtropical areas of the world. The largest endemic area in Europe can be found in the Po River Valley in northern Italy, where the prevalence in non-preventive-treated dogs ranges up to 80% [2]. *D. immitis* emerges in new countries due to globalization and increased travel as well as the import of infected dogs. In addition, climate change and the adaptability of vectors play major roles in the spreading of *D. immitis* [4]. Between 2014 and 2018, the number of imported *D. immitis* cases in dogs more than doubled, and it is suspected that Austria is facing pre-endemic status [5]. In Austria two mosquito species, *Aedes vexans* and the *Culex pipiens* complex are already known to act as potential vectors for *Dirofilaria* parasites. One main reason for the delayed introduction and establishment of *D. immitis* is the lack of microfilaraemic dogs as a consequence of less common kennelling or outdoor keeping of dogs in Austria [6]. Prevalence of feline heartworm infections is generally considered to be five to 20% of the canine counterpart population in the affected area [7].

Cats represent imperfect hosts for *D. immitis*. Compared to dogs, only a low number of L3 larvae develop to the adult stage, which also takes about seven to nine months. What is more, microfilariae (L1 larvae) are able to develop in only 20% of cats with mature female and male worms. Unlike in cats, significant microfilaraemia that can last for years develop in dogs. The lifetime of adult heartworms in cats up to four years old is shorter than in dogs, and adult *D. immitis* in cats are also smaller. Moreover, about 25% of cats are naturally resistant to infestation with *D. immitis* [2,7]. Cats with outdoor access seem to have a three-fold higher risk of being antigen-positive and male cats have been found to be more likely to develop mature infections [8]. On the other hand, heartworm disease as a differential diagnosis in indoor cats cannot be ruled out, but it is less likely [7]. Further proof that cats are imperfect hosts is the aberrant migration in body cavities, systemic arteries and the central nervous system, which occurs more frequently in cats than in dogs [2,7].

Severe pathological and life-threatening changes despite low parasite load of one to six adult worms per cat can be found early in cats [9]. After inoculation with L3 larvae they develop to Stage L4 and migrate to the pulmonary arteries 70–90 days post infection. The first phase is characterized by an intense eosinophilic pulmonary reaction. Most of the L4 die in this stage of disease and this stage is often misdiagnosed as feline asthma or chronic bronchitis, although this intense reaction is part of heartworm associated respiratory disease [10]. Sudden death in 20% of infected cats can be due to excessive inflammatory and thromboembolic response and is accompanied by haemothorax resulting from pulmonary artery dissection [10,11,12,13]. Caval syndrome is quite rare in cats, but it usually arises when one or two worms are located in the right heart causing tricuspid regurgitation. Most cats show moderate to mild symptoms, but owners also report chronic vomiting, anorexia and/or cachexia and respiratory signs [14].

To diagnose FHWD, a multimodal approach is necessary. A combination of diagnostic tools like thoracic radiographs, serum antibody tests, echocardiography and serum antigen tests are recommended. Necropsy is the gold standard for detecting adult worms [1,10,11]. Microscopic detection of microfilariae and ELISA to detect circulating antigens have low sensitivity in cats [1]. The Knott’s test for detecting circulating microfilariae is less successful, but when present, the FHWD diagnosis is confirmed [13].

In contrast to dogs, adulticidal therapy is not recommended because FHWD self-cures in most cases within 18–48 months. Surgical removal of the adult filariae can be performed in symptomatic cats. It is important to remove intact worms to avoid anaphylaxis. Monthly administration of macrocyclic lactones is strongly recommended in endemic areas [12,15].

To our knowledge, there are no reports of FHWD in Austria. In this case report, we describe a clinical case of autochthonous heartworm infection in a cat in Austria.

## 2. Case Report

**Figure 1:**
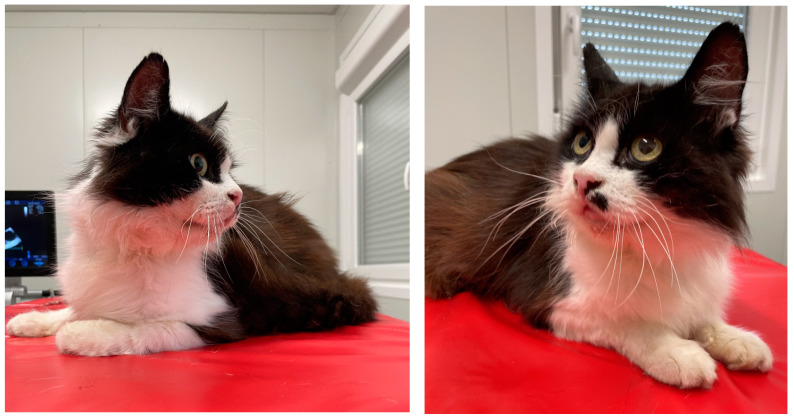
Male neutered European Shorthair cat referred for echocardiography.

A male neutered European Shorthair cat, seven years of age, 6 kg weight, was referred to the Veterinary Hospital Parndorf in the province of Burgenland, Austria for echocardiography in March 2020 (Figure 1). Prior to admittance, the cat had a short history of dyspnoea and increased abdominal circumference. At this time, the cat was the only animal in the household and had unrestricted outdoor access. The cat was in possession since kitten age, spending its lifetime in the same area, which is 47°34′57.443″ N, 16°32′33.673″ E. The cat was neither regularly vaccinated nor dewormed and had no history of periods abroad.

On clinical examination the cat showed calm and attentive behaviour, slightly pale mucous membranes, preserved skin elasticity, increased breathing, a systolic heart murmur Grade IV/VI best heard on the right hemithorax, moderate vesicular breath sounds, weak femoral pulse and lymph nodes within normal limits.

The pretreatment by the referral vet included Benazepril 0.4 mg/kg once daily and Furosemide 1.6 mg/kg twice daily with no improvement of clinical signs. The referral vet also took thoracic and abdominal radiographs. The thoracic radiographs showed a diffuse bronchointerstitial pattern of the pulmonary parenchyma with enlarged pulmonary arteries. The abdominal radiographs revealed decreased delimitation of the abdominal organs—most likely due to ascites.

### 2.1. Echocardiography

**Figure 2:**
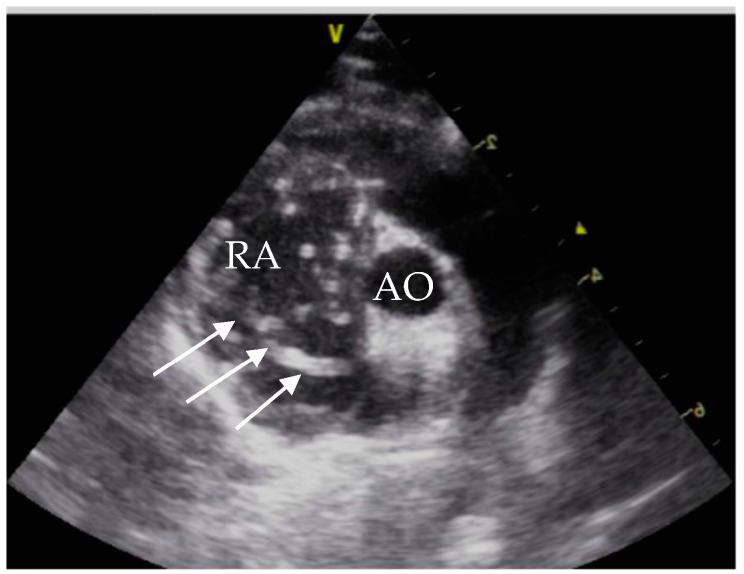
Echocardiography showing heartworms, right parasternal short axis view. RA = right atrium; AO = aorta; white arrows = heartworms.

Echocardiographic exams were performed by a single experienced operator with GE vivid™ S6, equipped with a microconvex GE 7s probe. The right atrium showed severe dilatation, and several double lined hyperechoic echoes close the tricuspid valve were seen (Figure 2). The suspected diagnosis based on the echocardiographic findings and the clinical signs was an infection with *D. immitis*.

After a period of reflection, the owner opted for a surgical attempt. Surgical removal of the suspected heartworms was scheduled two days after first presentation.

### 2.2. Complete Blood Count and Blood Chemistry

Before anaesthesia, blood analysis (CBC, biochemistry shown in Table 1) was performed via Idexx Procyte DX^®^ (Idexx Laboratories, Westbrook, ME, USA) and Idexx Catalyst DX^®^ (Idexx Laboratories, Westbrook, ME, USA). The cat showed a severe hyperchromic, macrocytic regenerative anaemia. What is more, the cat showed a mildly increased count of leucocytes with mildly increased neutrophils, lymphocytes and monocytes. Creatinine was 2.4 µmol/L which was in the upper reference interval. The Combo+ Snaptest^®^ (Idexx Laboratories, Westbrook, ME, USA) for infection with feline immunodeficiency virus (FIV) and feline leukemia virus (FeLV) showed a positive result for FeLV. No antigen testing for *D. immitis* was done before surgery due to the owner’s cost restrictions.

### 2.3. Surgical Removal of D. immitis

**Figure 3:**
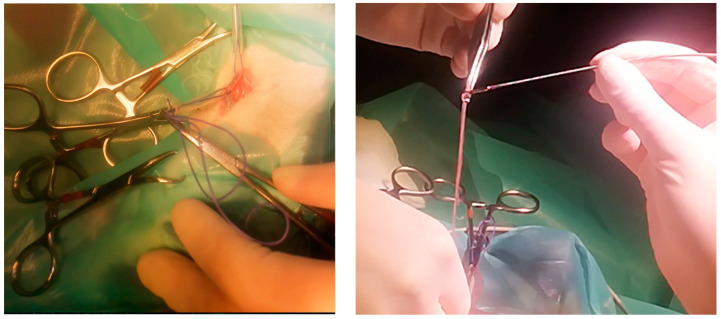
Surgical removal of the *Dirofilaria* specimens.

The cat was placed in left lateral recumbency for surgery. The right jugular vein was incised for a transvenous approach to the vena cava. A pair of flexible alligator forceps with 40 cm length—which usually finds use in endoscopy—was used to remove the suspected heartworms guided by echocardiography and C-arm. By use of this technique, which was described by Glaus et al., 1995 [16] the successful removal of 20 adult worms was possible (Figure 3, Video S1). Echocardiography revealed two to three dirofilariae left in the right atrium. After several subsequent attempts the right jugular vein was blocked, and removal of the remaining worms failed. The right jugular vein was ligated afterwards with a monofilament, absorbable suture material (Monosyn^®^ 3/0, Braun^®^) and the skin incision was also closed with the same suture material in a routine manner.

### 2.4. Identification of Dirofilaria Specimens

*Dirofilaria* specimens were stored in formalin for a year. DNA extraction of the middle piece of three specimens was performed with the DNeasy Blood and Tissue kit (Qiagen, Hilden, Germany) according to the manufacturer’s instructions. Briefly, tissue was homogenized in 180 µL buffer and three 2.8 mm ceramic beads (Precellys Ceramic Beads, Peqlab Biotechnologie GmbH, Erlangen, Germany) with a TissueLyser II (Qiagen, Hilden, Germany) for three minutes. Overnight digestion with 20 µL of Proteinase K was done at 56 °C. Buffer and ethanol were added and centrifuged in a Minispin column. After two washing steps, the DNA was collected with an elution buffer. Additionally, DNA was extracted with the QIAamp DNA FFPE Tissue kit (Qiagen, Hilden, Germany) according to the manufacturer’s guidelines. Tissue was rehydrated with 200 µL of 99.9% alcohol and subsequent centrifugation. The supernatant was discarded and 200 µL of 70% alcohol was added following centrifugation and discarding of the supernatant. Remaining alcohol was removed through evaporation and tissue was digested overnight with 180 µL buffer and 20 µL proteinase K at 56 °C. After incubation at 90 °C for one hour buffer and ethanol were added and centrifuged in a Minispin column. After two washing steps, the DNA was collected with an elution buffer. Amplification of the extracted DNA was performed with two different PCR protocols. Primers H14FilaCOIFw and H14FilaCOIRv targeted a 724 bp fragment and primers DI COI-F1 and DI COI-R1 targeted a 203 bp fragment of the mitochondrial cytochrome c oxidase subunit I gene and are published along with the PCR conditions elsewhere [17,18]. To improve amplification results, PCRs were repeated with the GoTaq^®^ Long PCR Master Mix (Promega, Madison, WI, USA). PCR products were separated in 2% agarose gels stained with Midori Green Advanced DNA stain (Nippon Genetics Europe, Düren, Germany) by electrophoresis. Amplification of a 203 bp or 724 bp fragment of the cytochrome c oxidase subunit I gene was unsuccessful.

**Figure 4:**
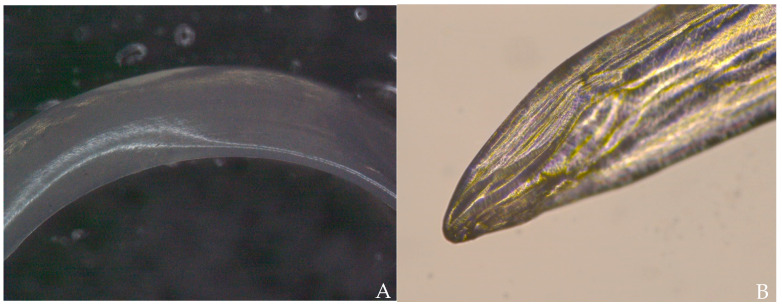
(**A**) Stereomicroscopic image of the body of adult D. immitis. The cuticle is smooth and does not have longitudinal cuticular ridges as could be found in adult D. repens. (**B**) Light microscopic image of the cephalic extremity of adult D. immitis.

**Figure 5:**
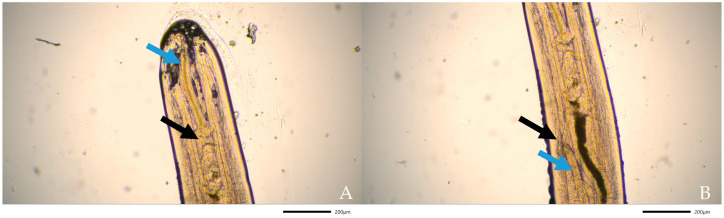
(**A**) Light microscopic image of cephalic extremity adult *D. immitis*. The oesophagus (blue arrow) and intestine (black arrow) are visible. (**B**) Light microscopic image of adult female *D. immitis*. Vulvar opening (black arrow) and uterus (blue arrow) are visible.

**Figure 6:**
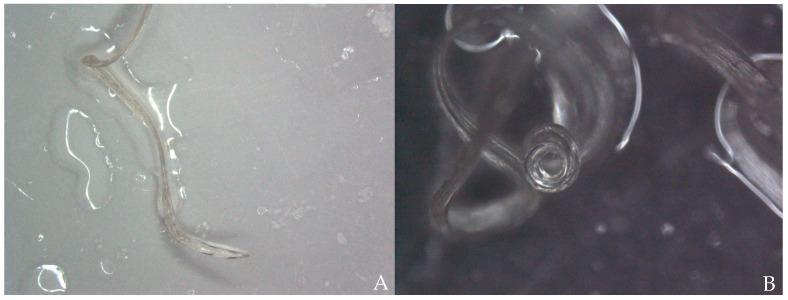
(**A**) Stereomicroscopic image of the straight tail of adult female *D. immitis*. (**B**) Stereomicroscopic image of the tail of adult male *D. immitis*. The tail in males is wound up in a corkscrew manner.

Using light microscopy (Nikon Eclipse Ci, Tokyo, Japan) and stereo microscopy (Nikon SMZ1270, Tokyo, Japan) five specimens were analysed of which three were female and two were male. Female specimens were 212 to 250 mm long and 0.74 to 1.08 mm wide. Male specimens were 97 to 116 mm long and 0.63 to 0.66 wide; however, in one specimen, the tail was missing. The cuticle was smooth and lacking significant longitudinal cuticular ridges (Figure 4A), showing cephalic extremity slightly thin and rounded (Figure 4B), no clear demarcation between the oesophageal muscular and glandular regions (Figure 5A), visible uterus (Figure 5B) and straight tail in females (Figure 6A) and tail wound-up in a corkscrew manner in males (Figure 6B). All morphological features were compatible with adult stages of *D. immitis*. 

### 2.5. Postoperative Course

After surgery, the cat’s creatinine increased to 5.1 mg/dL and the haematocrit decreased to 12.8%—but showed still high regeneration with a reticulocyte count of 158.7 G/L. It was suspected that the cat had an episode of acute kidney failure due to the removal of the heartworms. The therapy included buprenorphine 10 µg/kg three times a day, amoxicillin–clavulanic acid 20 mg/kg twice daily, doxycycline 5 mg/kg twice daily, spironolactone 2 mg/kg twice daily, pimobendan 0.25 mg/kg twice daily, and calcium carbonate to lower the phosphorus level. Fortunately, the cat’s medical condition improved, and it was sent home three days after surgery with a creatinine of 3.2 mg/dL and a stable haematocrit of 12%.

The prescribed treatment included pimobendan 0.25 mg/kg twice daily, clopidogrel 1.5 mg/kg once daily and milbemycin 4 mg/kg monthly. Further control examinations were performed at the referral vet.

### 2.6. Follow Up

**Figure 7:**
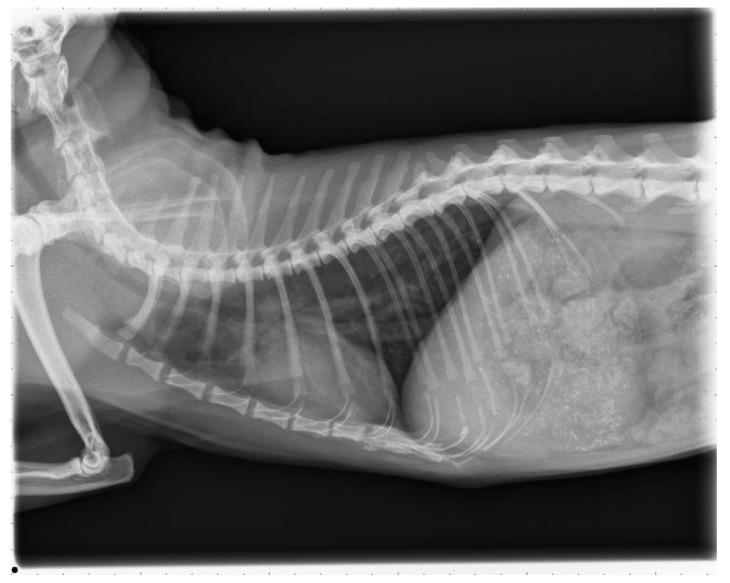
Thoracic radiograph, right laterolateral view.

The cat was presented 16 months after surgery for a control examination including thoracic radiographs and echocardiography at the Veterinary Hospital in Parndorf, Burgenland. The owner reported that the cat was not showing any clinical signs despite of infrequent, spontaneous coughing and receiving the prescribed medication daily. Clinical examination remained unremarkable. The thoracic radiographs showed a moderate bronchointerstitial lung pattern without enlarged pulmonary arteries (Figure 7). This bronchointerstitial lung pattern could be due to heartworm associated respiratory disease (HARD).

**Figure 8:**
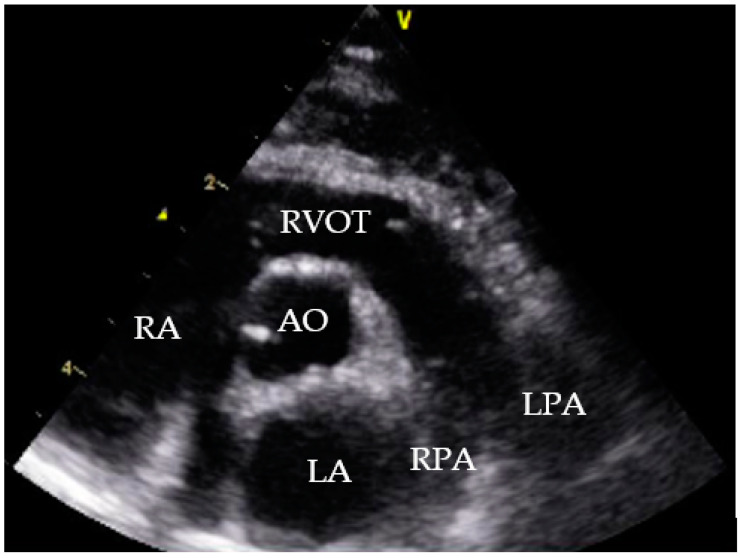
Echocardiogram July 2021, right parasternal short axis view. RA = right atrium; RVOT = right ventricle outflow tract; AO = aorta; LA = left atrium; RPA = right pulmonary artery, LPA = left pulmonic artery.

Additionally, an echocardiography was performed. The cat showed a tricuspid insufficiency (2.8 m/s), a mild mitral insufficiency and a pulmonary artery flow of V_max_ 1.1 m/s (probably suggesting mild pulmonary hypertension). Comparing the two echocardiograms the cat’s hemodynamic status significantly improved (Table 2) with increased left ventricle preload. No heartworm was detectable (Figure 8). LA/Ao could not be measured in the initial echocardiography due to the enlarged right atrium.

Complete blood count and creatinine were repeated, and CBC showed no abnormalities with a haematocrit of 30.5% and creatinine of 1.9 mg/dL (Table 1).

What is more, the Knott’s test to detect microfilaria was negative. In addition, the Heartworm-Snap-Test^®^ (Idexx Laboratories, Westbrook, ME, USA) showed a negative result. Felichek-3^®^ (Bionote), a chromatographic immunoassay showed negative results for feline heartworm antibodies as well as feline immunodeficiency virus antibody and feline leukaemia virus antigen.

At this time, in July 2021, there was no sign of reinfection with *D. immitis* in this cat.

## 3. Discussion

*Dirofilaria immitis* has shown a rapidly increasing prevalence over the past few years, especially in Central Europe. FHWD can be detected in the same areas as canine heartworm disease at up to 20% of the rate in unprotected dogs [7,19]. The cat in this case report lives in Horitschon, which is approximately 4.5 km linear distance from the Hungarian border. Due to climate change and abundance of mosquito vectors, the development and transmission of *D. immitis* and *D. repens* has increased over the past few years and Hungary now also belongs to one of the endemic areas in Europe [20]. Male cats are reported to have a larger home range than female cats. It is reported that desexing male cats should decrease their home range, because their behaviour will be focused more on foraging than mating [21]. In a study with fourteen house cats, the home range for wandering cats was 5.1 ha. The longest linear distance travelled by a house cat in this study was 1.17 km. In another study from 2015, the home range was between 2.66 and 5.52 ha for free-ranging farm cats. One study of 2020 including 925 cats, only three cats exceeded the usual home range of less than 1 km^2^ [22,23,24]. In this case report we cannot completely rule out that the cat crossed the Hungarian border and got infected there or that mosquitos crossed the border and infected the cat at its home farm, even if it seems improbable. As part of a study showing the incidence for *D. immitis* in shelter dogs and mosquitoes, 205 mosquito species were trapped in Austria and 115 dogs were tested for *D. immitis* infection. Forty-six of these mosquitoes were found in Burgenland. In none of these 205 mosquitoes has DNA of *D. immitis* been found to date but several dogs in a local shelter, all originating from Hungary, tested positive for *D. immitis* [25].

At initial presentation, the cat showed dyspnoea, ascites and double hyperechoic parallel lines in the echocardiography, which is the typical presentation for *D. immitis* in echocardiography [26]. The sensitivity of echocardiography for detection of *D. immitis* is operator-dependent and is reported between 88% and 100%. False positive results can be caused by right ventricular chordae tendineae [27].

As a differential diagnosis, *Angiostrongylus chabaudi* was assumed. At necropsies, immature nematodes of *A. chabaudi* can be found in the pulmonary arteries without evidence of L1 in faeces [28].

*A. chabaudi* was first described in 1957 in a wild cat in Central Italy. More case reports of cats with *A. chabaudi* in Greece, Romania, Italy, Bulgaria, Bosnia-Herzegovina and Germany do exist but it is not known yet if angiostrongylosis caused by *A. chabaudi* is clinically relevant or not. Pathological lesions like granulomatous pneumonia, hyperplasia of pulmonal arteries and thrombosis are reported [29]. In our case report we assume that FHWD is the most likely diagnosis due to clinical presentation and findings in echocardiography. Currently no publications exist regarding *A. chabaudi* being detectable in echocardiography. In dogs, *Angiostrongylus vasorum* cannot be seen in echocardiography, so it also seems unlikely that *A. chabaudi* can be seen in echocardiography [30].

Another differential diagnosis with emphasis on the radiographic changes are infections with *Aelurostrongylus abstrusus* and *Troglostrongylus brevior*. The most common signs of aelurostrongylosis are dry or productive cough, dyspnoea, tachypnoea as well as weight loss, anorexia and fever. Pleural effusion or pneumothorax caused by *A. abstrusus* can lead to death. Secondary pulmonary hypertension is caused by the local inflammation triggered by parasite stages. Clinical presentation of cats with *T. brevior* is similar, although the nematode seems to be more pathogenic in kittens and young animals. Due to the paucity of clinical studies on this disease, knowledge on the radiographic features of troglostrongylosis is still poor [31,32]. To our knowledge, no publications exist regarding *A. abstrusus* and *T. brevior* being detectable in echocardiography, what makes these differentials unlikely.

The cat also showed right atrial as well as right ventricular enlargement, which underlined our suspected diagnosis of FHWD. These findings seem to appear quite rarely in cats and are commonly observed in dogs [1]. What is more, the cat showed diffuse bronchointerstitial lung pattern and enlarged pulmonary arteries in the thoracic radiographs, which were taken by the referral vet. These findings in the radiographs are also described as common changes in cats with FHWD although the diffuse parenchymal pattern can also occur in cats with asthma or aelurostrongylosis [33]. In this case report, no test for microfilaraemia was performed during initial diagnosis. Even using special techniques like the Knott’s test microfilaraemia is only detected in less than 20% of cats with adult heartworms. When microfilariae are present, it is considered a definitive diagnosis for FHWD [34]. Antigen testing is still considered the gold standard. One disadvantage of serological testing is that antibodies and antigen circulate for an indeterminate length of time after the parasite has been cleared [35]. Antibodies against *D. immitis* are found two months post infection. False positive test results as consequence of clearing the infection can be found as well as false negative results in asymptomatic cats. Running antigen and antibody tests can improve the sensitivity compared to running one test alone [15,34]. In our case report, neither an antigen nor antibody test was performed due to the indication of FHWD in echocardiography and thoracic radiographs.

Usually, the worm burden is low and infections with only male or female adults reduce the sensitivity of the antigenic reaction. Detection of antigen cannot confirm the presence of immature stages of the parasite. A negative antigenic test cannot be the basis for ruling out an infection with *D. immitis*. Therefore, the result should be recorded as “no antigen detected” [1,10]. In this case, the cat’s worm burden with 20 removed adult heartworms was unusually high. As part of a study, cats were experimentally infected with 100 L3 larvae, three to ten adult heartworms developed in 75% of the cats in this study population [1]. This high worm burden could either be due to multiple bites by infected mosquitoes or decreased immune response, as the cat was seropositive for FeLV. One study showed no association between heartworm infection and co-infection with FIV or FeLV. Male uncastrated cats had a higher risk of infection with heartworm, FeLV and FIV than females. Another study from 2017 found that cats with retroviral infections—especially FIV—had a marked increase of seropositivity for *D. immitis*. They postulate that this is not necessarily related to a relationship with heartworm infection but might be due to common predisposing factors, such as outdoor roaming. Contrary to this study, no apparent correlation with FeLV and FIV infection was noted in a study of 2011 [8,20,34]. In addition, the cat showed negative results for FeLV 16 months after surgery. This could be due to an abortive or regressive infection [36]. No further testing for FeLV provirus was done. To our knowledge, a worm burden as high as was found in our subject has not been reported before.

The cat showed also severe hyperchromic, macrocytic regenerative anaemia. Anemia is described in cats and dogs with caval syndrome due to haemolysation [37].

In this case, we decided to remove the adult nematodes surgically. Acute death of cats can occur when even only one worm is present [1,10]. In most cases, the prognosis for caval syndrome is poor, so surgical removal seemed the only realistic chance for this cat. Different authors have described techniques for the removal of heartworms. We decided to use a transvenous approach through the right jugular vein. A limiting factor of this technique can be the body size of small cats. Major complications range from iatrogenic damage that results in thrombus formation, damage to the endo- or myo-cardium, tricuspid valve or chordae tendinae as well as iatrogenic damage that cause pneumothorax [37,38]. If a transvenous approach is not possible, right atriotomy using total venous inflow occlusion is prescribed. One advantage of this technique is the in-situ removal of heartworms, whereas removal with alligator forceps can break the heartworms and cause a shock-like reaction induced by the worm’s body fluid. In addition, main pulmonary arteriotomy as well as right auriculotomy are described as therapy for cats with caval syndrome. The cat developed acute kidney failure postoperatively. Hepatorenal dysfunction is also reported in cats with *D. immitis*. It is associated with poor tissue perfusion and hyporexia of these organs. In necropsy of dogs with caval syndrome tubular necrosis and haemosiderosis was found [14,35,38].

DNA extraction of formalin fixed tissue is challenging, and usually only short fragments can be amplified. A study comparing two DNA extraction kits showed better results with the QIAamp DNA FFPE Tissue kit (Qiagen, Hilden, Germany), which is designed for DNA extraction from formalin fixed paraffin embedded tissue but is recommended for the use of tissue that has been fixed in formalin no longer than 24 h. In this study, up to 171 bp sequences could be amplified from historic formalin fixed tissue, albeit with low purity [39]. Considering this outcome, we did not expect successful amplification of the 202 bp sequence and did not attempt to extract DNA from the formalin fixed tissue any further. Recently published, more advanced protocols might overcome these limitations, but do require special equipment [40]. A definitive morphological identification was possible and comparable to other publications [41].

After surgery, the cat was treated with doxycline 5 mg/kg twice daily orally to target *Wolbachia*. *Wolbachia* spp. plays an important role in the survival of filarioid nematodes and gets amino acids for bacterial growth in turn. In dogs, pre-treatment with doxycycline before adulticidal therapy helps to reduce pulmonary pathology. In cats, this benefit has not yet been evaluated. That is the reason why doxycycline is not recommended as an adjunctive therapy in cats at the moment [1,34]. Clopidogrel was prescribed to prevent thromboembolism although there is lack of evidence in literature. In asymptomatic cats adulticidal therapy is not recommended due to the self-limiting infection within 18–48 months. Melarsomine, which is used as adulticidal therapy in dogs is not safe in cats and can trigger pulmonary thromboembolism and anaphylactic reactions as result of parasite death. Melarsomine is toxic to cats at doses as low as 3.5 mg/kg [1,10,15].

To prevent reinfection, the owner was advised to give milbemycin 4 mg/kg monthly following surgery. Monthly chemoprophylaxis is recommended from eight weeks of age year-round to kill L3 and L4 larvae. Ivermectin and milbemycin oxime, both administered orally, as well as topical moxidectin and selamectin can all be used for the prevention of FHWD [15].

Considering the lack of clinical signs and detectable abnormalities at clinical, sonographic and laboratory examination at 16 months post-surgery, the cat can be considered fully recovered. Coughing can be a long-term effect of HARD. Parasite death can be associated with severe pulmonary thromboembolism and eosinophilic inflammatory response in the lungs, causing HARD. Chronic, histologic evident myofibrocyte proliferation can be observed up to 18 months after infection [42].

In a study with asymptomatic cats with HWD, it was quite impossible to predict the outcome if the infection was diagnosed early. Three cats in this study died suddenly after 38–40 months post-diagnosis. In addition, if the duration of infection from diagnosis to death exceeds 1000 days, it is too long to implicate HWD as cause of death. Another retrospective study of symptomatic cats showed a median survival time of 1.5 years overall [43,44]. Our cat in this case report now nearly exceeds this median survival time.

The spreading of *D. immitis* worldwide due to climate change, globalization and increased travel of infected dogs is the reason Austria is facing the pre-endemic status [5,6]. This case report shows the first cat in Austria with an autochthonous *D. immitis* infection. Austrian veterinarians should be aware of the zoonotic potential of *D. immitis*. Increased and more intensive communication about prevention with owners living in close owner-pet-relationships is necessary. FHWD should be considered as a differential diagnosis if cats are living in border regions of surrounding countries. Macrocyclic lactones should not be used only for deworming (e.g., milbemycin oxime) but also for prevention of HWD.

## 4. Conclusions

The spreading of *D. immitis* worldwide due to climate change, globalization and increased travel of infected dogs is the reason Austria is facing pre-endemic status [5]. This case report describes what is potentially the first documented autochthonous *D. immitis* infection in Austria. In our case, a cat was infected with *D. immitis*. Furthermore, considering that the cat is not the natural reservoir of the parasite, it must be deduced that in the same area there were dogs infected by *D. immitis* and not diagnosed. Increased and more intensive communication about prevention with owners living in close owner-pet-relationships is necessary. FHWD should be considered as a differential diagnosis if cats are living in border regions of surrounding countries. Macrocyclic lactones should not be used only for deworming (e.g., milbemycin oxime) but also for prevention of HWD.

## Figures and Tables

**Table 1:** CBC and blood chemistry—March 2020 and July 2021.

Parameter	Value		From	To	Measuring Unit
	March 2020	July 2021			
Erythrocytes (RBC)	2.72	7.75	6.54	12.20	T/L
Haematocrit (HCT)	12.0	39.50	30.30	52.30	L/L
Haemoglobin	5.20	9.90	9.80	16.20	mmol/L
MCV	44.10	39.40	35.90	53.10	fL
MCH	19.10	12.80	11.80	17.30	fmoL
MCHC	43.30	32.50	28.10	35.80	mmol/L
Reticulocytes	168.40	10.90	3.0	50.0	G/L
Leukocytes (WBC)	19.10	11.80	2.87	17.02	G/L
Neutrophils	15.68	8.47	2.30	10.29	M/L
Lymphocytes	1.91	1.93	0.92	6.88	M/L
Monocytes	0.94	0.42	0.05	0.67	M/L
Eosinophil	0.54	0.95	0.17	1.57	M/L
Thrombocytes (PLT)	175	594	151	600	GL
Creatinine	2.4	1.9	0.8	2.4	mg/dL

**Table 2:** Echocardiograms compared (March 20 to July 21).

Date	IVSd	LVIDd	LVPWd	LA/Ao
4 March 2020	0.58 cm	1.20 cm	0.60 cm	-
14 July 2021	0.55 cm	1.75 cm	0.42 cm	1.35

IVSd = interventricular septal end diastole, LVIDd = left ventricular internal diameter end diastole, LVPWd = left ventricular.

## Data Availability

Not applicable.

## Supplementary Materials

The following are available online at https://www.mdpi.com/article/10.3390/pathogens10091104/s1, Video S1: Surgical removal of *Dirofilaria immitis*.
